# Characterization of Traumatic Brain Injury in a Gyrencephalic Ferret Model Using the Novel Closed Head Injury Model of Engineered Rotational Acceleration (CHIMERA)

**DOI:** 10.1089/neur.2023.0047

**Published:** 2023-11-09

**Authors:** Justin L. Krieg, Anna V. Leonard, Renee J. Tuner, Frances Corrigan

**Affiliations:** Translational Neuropathology Laboratory, School of Biomedicine, Faculty of Health and Medical Sciences, The University of Adelaide, Adelaide, Australia.

**Keywords:** CHIMERA, diffuse axonal injury, ferret, gyrencephalic model, traumatic brain injury

## Abstract

Traumatic brain injury (TBI) results from mechanical force to the brain and leads to a series of biochemical responses that further damage neurons and supporting cells. Clinically, most TBIs result from an impact to the intact skull, making closed head TBI pre-clinical models highly relevant. However, most of these closed head TBI models use lissencephalic rodents, which may not transduce biomechanical load in the same manner as gyrencephalic humans. To address this translational gap, this study aimed to characterize acute axonal injury and microglial responses in ferrets—the smallest gyrencephalic mammal. Injury was induced in male ferrets (*Mustela furo*; 1.20–1.51 kg; 6–9 months old) with the novel Closed Head Injury Model of Engineered Rotational Acceleration (CHIMERA) model. Animals were randomly allocated to either sham (*n* = 4), a 22J (joules) impact (*n* = 4), or a 27J impact (*n* = 4). Axonal injury was examined histologically with amyloid precursor protein (APP), neurofilament M (RMO 14.9) (RMO-14), and phosphorylated tau (AT180) and the microglial response with ionized calcium-binding adaptor molecule 1 at 24 h post-injury in gray and white matter regions. Graded axonal injury was observed with modest increases in APP and RMO-14 immunoreactivity in the 22J TBI group, mostly within the corpus callosum and fornix and more extensive diffuse axonal injury encompassing gray matter structures like the thalamus and hypothalamus in the 27J group. Accompanying microglial activation was only observed in the 27J group, most prominently within the white matter tracts in response to the larger amounts of axonal injury. The 27J, but not the 22J, group showed an increase in AT180 within the base of the sulci post-injury. This could suggest that the strain may be highest in this region, demonstrating the different responses in gyrencephalic compared to lissencephalic brains. The CHIMERA model in ferrets mimic many of the histopathological features of human closed head TBI acutely and provides a promising model to investigate the pathophysiology of TBI.

## Introduction

Traumatic brain injury (TBI) is the result of external force being applied to the head, accounting for 30% of all injury-related deaths.^1^ Of these, the majority are classified as mild TBI or concussion,^2^ although even with mild TBI up to half of patients remain symptomatic up to 12 months post-injury.^3^ Though less common, moderate-severe TBI is associated with longer periods of hospitalization and can require extensive rehabilitation, placing a large burden on healthcare systems.^4^

TBI can be the result of a focal, direct load to the head that produces focal injury on impact or by inertial forces causing rapid head displacement with more diffuse pathology. Rapid tissue deformation from TBI causes damage to vasculature, axons, and parenchyma, known as primary injury.^5^ This sets off a biochemical response encompassing alterations in cerebral blood flow, inflammation, axonal injury, calcium influx, and oxidative stress, among others, known as the secondary injury cascade, which further exacerbates damage to the parenchyma, leading to worsened outcomes clinically.^5–7^ Axonal injury is thought to drive many of the functional deficits observed after both mild^2^ and moderate-to-severe TBI.^8^ Most axonal injury is not caused by the primary insult, but attributable to the initiation of secondary injury processes. Cytoskeletal elements, such as the microtubules, appear to be particularly vulnerable to mechanical forces,^9,10^ with areas of microtubule disruption leading to interruption of axonal transport and accumulation of axonally transported proteins.^11^ Additionally, mechanical force can result in mechanoporation and subsequent calcium influx, thereby exacerbating cytoskeletal disruption through activation of calpains.^12^

Pre-clinical models are essential in understanding how axonal injury develops post-TBI. Closed head diffuse impacts are the most common clinical forms of TBI; therefore, models reproducing this injury type are highly relevant for the characterization of axonal injury.^13^ Brain structure is a key consideration for brain injury models, given that the presence of gyri alters the response to the impact, with the presence of gyri and sulci concentrating the force within the cortex by 2.8-fold.^14^ Non-human primates, sheep, and pigs are all suitable candidates, though the high costs and resources, attributable to the specialized requirements for husbandry, surgery, and functional assessments, may be prohibitive.^15,16^ Ferrets, being the smallest gyrencephalic mammal, may help to overcome these obstacles. Ferrets possess a high degree of white matter compared to rodents and have subcortical short association fibers associated with high-order cognition, alongside a ventral hippocampus.^17,18^

Additionally, cortical thickness and layer distribution are more akin to humans in the ferret compared to rodents.^19^ These anatomical features in ferrets, which are more akin to the human brain, when paired with gyrencephaly, make them an ideal model for studying the effects of TBI. Indeed, ferrets have been used in both focal^20–22^ and blast injury models,^23,24^ resulting in chronic white matter inflammation, akin with human TBI.^21^ However, both focal and blast TBI differ in key aspects of the secondary injury response within the brain.^25,26^ Accordingly, developing a purely diffuse injury model would be advantageous to potentially characterize various clinical TBI types.^25^

The CHIMERA (Closed Head Injury Model of Engineered Rotational Acceleration) allows a reproducible, controlled closed head TBI and has been extensively characterized in mice and rats.^26–30^ Additionally, this model is designed to provide a high degree of diffuse axonal injury (DAI) without producing contusions. A preliminary report by Hutchinson and colleagues demonstrated the feasibility of the model in ferrets, but histological evaluation of the effects of single closed head TBI has yet to occur.^31^ Here, we examined the acute spatial distribution of axonal injury and the accompanying microglial response to a CHIMERA impact delivered at either 22 or 27J (joules) of input energy in male ferrets.

## Methods

### Experimental design

Studies were designed in accordance with guidelines from the National Health and Medical Research Committee of Australia, and procedures were approved by the South Australian Health and Medical Research Institute Animal Ethics Committee (SAM 20-041).^32^ Twelve male ferrets (*Mustela furo*; 1.20–1.51 kg; 6–9 months old) were group housed in modified rabbit cages, enriched with hammocks and toys, for at least a month before any procedures. Ferrets had *ad libitum* access to food and water and were on a 12 h light/dark cycle with room temperatures maintained between 18℃ and 24℃. Animals were assigned to sham (*n* = 4) or two TBI groups: 22J (*n* = 4) and 27J (*n* = 4), using a random number generator and transcardially perfuse fixed after 24 h.

### Animal preparation

Animals were induced using a feline induction box with 4% isoflurane (Henry Schein Animal Health, Dublin, OH) with 1 L/min of 100% oxygen for 5–7 min. After a loss of palpebral and pedal reflexes, animals were intubated using a non-cuffed 2.0–3.0 endotracheal tube and connected to a rebreathing circuit. Animals were maintained on 1–3% isoflurane with 1 L/min of 100% oxygen. The endotracheal tube was sutured to the top lip of the animal using a 3-0 suture with a cutting needle. Ferrets had continuous monitoring of O₂ saturation, expired CO₂ concentration, heart rate, blood pressure, respiratory rate by a pulse oximeter (MP30; Phillips, Amsterdam, the Netherlands), and a non-invasive blood pressure monitor (BZ-9000F; BZ Technology Co., Ltd., Beijing, China). Ferrets were kept warm using a fixed-temperature heat pad and warmed saline bags. Once anesthetized, ferrets were given 10 mL of 0.9% subcutaneous saline across two sites and a single dose of buprenorphine (0.015 mg/kg; Troy Laboratories, Glendenning, NSW, Australia) subcutaneously for post-operative analgesia.

### Injury

**FIG. 1.**
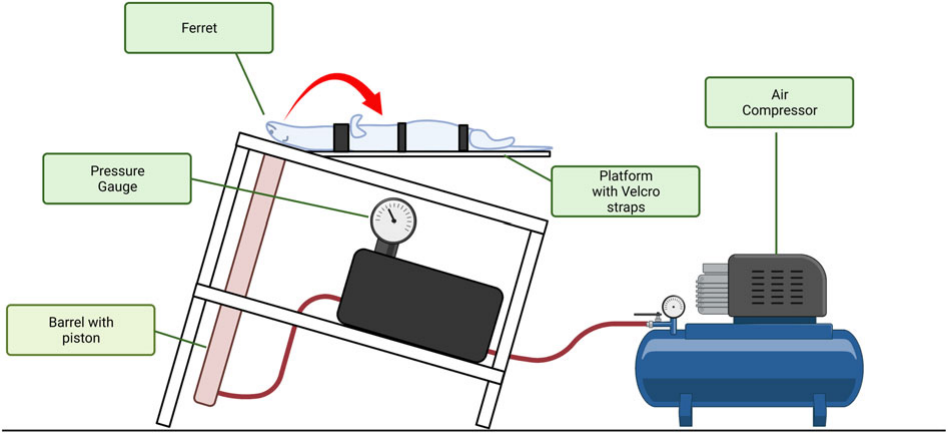
Schematic overview of the CHIMERA injury device, highlighting the ferret's position on the device and the major components. Briefly, compressed air is put into the chamber at a set PSI, and, upon computer activation, the piston fires upward toward the head of the animal. Once contact is made, the animal's head accelerates forward and contacts the sternum before returning to the baseline position. Figure generated with BioRender software (BioRender, Toronto, Ontario, Canada). CHIMERA, Closed Head Injury Model of Engineered Rotational Acceleration; PSI, pounds per square inch.

Injury was induced with the CHIMERA device, which uses a pneumatically driven 200-g impactor tip to make contact on the dorsal skull, as previously described in mice.^26^ The desired injury severity is modulated through air pressure within the system; hence, pounds per square inch (PSI) is proportional to the impact energy. We report joules and not PSI because this calculation accounts for the size of the device and hence standardizes input energy across different species. For this study, 22 and 27J were selected in alignment with previous ferret studies using the CHIMERA injury device.^31^ Ferrets were placed in a supine position on the CHIMERA device and their head was aligned with the crosshairs on the injury platform to ensure consistent injury location (Fig. 1). The body was secured with three Velcro straps, with the top strap located at the shoulder. Once the device was activated, the piston fired upward and contacted the head, causing the head to swing forward, contacting the sternum before returning to the platform. Ferrets were then returned to the heat pad. Sham animals were also placed on the injury device, aligned, and secured with Velcro straps, but the device was not fired.

Vital signs were monitored every 5 min post-TBI for 15 min total, with animals kept under anesthesia for 30 min post-TBI before they were recovered on 1 L/min of 100% oxygen. Animals were extubated upon the return of a coughing reflex and returned to their home cage once ambulant. Ferrets were monitored after the procedure for any signs of distress or grimacing such as orbital tightening, bed avoidance, and any other abnormal behavior. All ferrets were given a single serving of wet cat food to assist their recovery.

### Perfusion and tissue collection

**FIG. 2.**
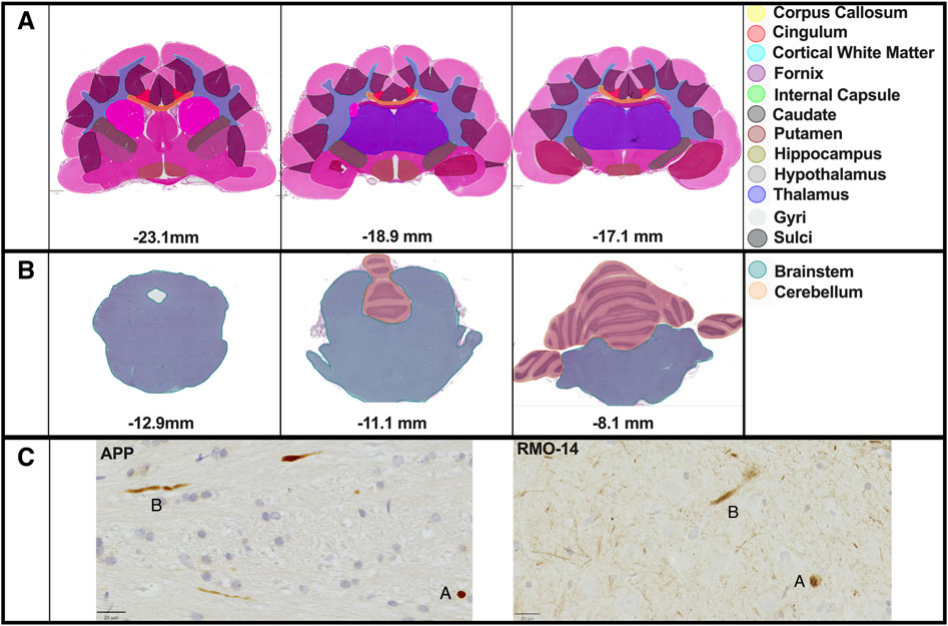
H&E outlines of the regions of interest across the cortex, white matter structures and subcortical structures (**A**), and brainstem (**B**). All stains were analyzed in all regions of interest. Representative staining of APP and RMO-14 DAI is shown in panel **C**. APP, amyloid precursor protein; DAI, diffuse axonal injury; H&E, hematoxylin and eosin; RMO-14, neurofilament M (RMO 14.9).

At 24 h post-injury, animals were reanesthetized and transcardially perfused with heparin (1000 IU; Pfizer, New York, NY), followed by 1 L of 10% neutral buffered formalin (POCD Scientific, North Rocks, NSW, Australia), and brains were subsequently harvested. Brains were assessed for any evidence of contusions macroscopically. Brains were then sectioned into 10-mm coronal slices, embedded in paraffin wax, and cut into 5-μm sections and mounted on slides (HistoBond; Paul Marienfeld GmbH & Co. KG, Lauda-Königshofen, Germany), in accordance with the ferret brain atlas.^18^ Bregma and lambda are scarcely observed in ferrets because of the development of a sagittal crest.^18^ So the occipital crest is used as a point of reference. Coronal sections were cut at −23.1, −18.9, and −17.1 mm relative to the occipital crest (Fig. 2A). These sections were selected to maximize the number of anatomical regions of interest, with three levels also in line with previous rodent CHIMERA characterization studies.^29^ To assess brainstem pathology, sections were cut −12.9, −11.1, and −8.1 mm from the occipital crest, giving visibility to the midbrain, pons, and cerebellum (Fig. 2B).

### Morphology

Hemoxylin and eosin (H&E) was used to assess the overall integrity of brain tissue and for evidence of hemorrhagic or ischemic lesions. Slides were cover-slipped and scanned using a Hamamatsu NanoZoomer 2.0RS (Hamamatsu Photonics K.K., Shizuoka, Japan) to obtain high-resolution images. Sections were assessed for macroscopic lesions using QuPath software (v0.3.2).

### Immunohistochemistry

Slides were dewaxed and immersed in methanol and 1.5% hydrogen peroxide to block endogenous peroxidase activity. Heat-induced epitope retrieval was done with citrate (pH = 6) to recover antigen reactivity. Blocking was done using normal horse serum (NHS; catalog no.: S-2000-20; Vector Laboratories, Newark, CA) for 30 min. Primary antibodies included APP (amyloid precursor protein; 1:2000; catalog no.: 51-2700, RRID:AB_2533902; ThermoFisherScientific, Waltham, MA) and (neurofilament M (RMO 14.9) (RMO-14; 1:8000; catalog no.: 34-1000, RRID:AB_2533154; ThermoFisherScientific) for axonal injury markers, AT180 (Phospho-PHF-tau; 1:250; catalog no.: MN1040, RRID:AB_223649; ThermoFisherScientific) for tau phosphorylation, and IBA-1 (ionized calcium-binding adapter molecule 1; 1:2000; catalog no.: 019-19741, RRID:AB_839504; Wako Chemicals USA, Richmond, VA) for microgliosis. Primary antibodies were applied and incubated overnight at room temperature. Negative control tissue received NHS only. Secondary antibodies (1:250; Vector Laboratories: anti-rabbit [catalog no.: BA-1100] or anti-mouse [catalog no.: BA-2000]) were applied for 30 min followed by a tertiary of horseradish peroxidase streptavidin (1:1000; SA-5004; Vector Laboratories) for 1 h.

Slides were washed with phosphate-buffered saline with 0.03% Triton X-100 in between each antibody application. Antigen visualization was done through the application of 3,3-diaminobenzidine (DAB; SK-4100; Vector Laboratories), before counterstaining in hematoxylin. Slides were cover-slipped and scanned.

### Physiological analysis

Pre-injury baseline data presented are the average of all measurements taken in the 15 min before injury induction. Data collection was non-discrete throughout the procedure; hence, physiological parameters were binned into 5-min allotments post-TBI and averaged up to 15 min post-TBI, capturing the immediate physiological response of the injury.

### Histological analysis

Primary analysis was conducted in tissue across three anteroposterior levels in either the forebrain or brainstem. Regions of interest in the cerebrum are summarized in Figure 2A. White matter structures included the corpus callosum, cingulum, cortical white matter, fornix, and internal capsule. Subcortical structures included the thalamus, hypothalamus, caudate, putamen, and hippocampus. The neocortex was divided into gyri and sulci. Full cerebrum counts were the sum of all regions of interest and any remaining tissue. Furthering this, brainstem pathology was assessed and included three anteroposterior levels between the midbrain and pons (Fig. 2B). All stains were first analyzed and averaged across these anteroposterior levels. QuPath was used to annotate and analyze APP, RMO-14, and phosphorylated tau.^33^ IBA-1 was analyzed using the Indica Labs HALO platform. Where appropriate, QuPath's mask image name function was used to blind investigators by obfuscating animal identifications with random characters. Unfortunately, because of technical errors, one 22J animal was trimmed through at the −23.1-mm section, leading to missing caudate and putamen values. Additionally, three cerebellum values are missing for the IBA-1 stain and one putamen value for IBA-1. Overall, this represents 11 missing values out of the reported 839 values.

#### Amyloid precursor protein and neurofilament M (RMO 14.9)

Both APP and RMO-14 immunoreactivity was assessed using QuPath's point annotation tool to manually count APP-positive or RMO-14^+^ axons with the expression of counts as positive axons/mm^2^ per region of interest, including both transverse dot-like and in-plane longitudinal axons (see Fig. 2C).

In addition to region quantification, spatial distribution was assessed using QuPath's generate density map functions. This generated graded color maps in accordance with the density of DAI. These density maps are defined by putting a 250-μm radius around each detection and, for the degree of overlap with other detections, informs the mapping color, ranging from black, where no detections overlap, to a bright yellow, where 50 injured axons overlap in this small radius. Parameters were chosen that best represented the distribution of axonal injury across severities.

#### Phosphorylated tau

AT180 immunoreactivity was assessed using QuPath's positive pixel thresholder function (resolution, full; channel, DAB; smoothing sigma, 0; threshold, 0.7), which identified the area of staining that was above the DAB threshold.

#### Ionized calcium-binding adapter molecule 1

IBA-1 sections were analyzed using the Indica Labs HALO platform (v3.0.311.360), using the microglial activation extension (v1.4). This allowed for semiautomated microglial counting and the analysis of activation states (cell diameter, 1.66; contrast, 0; optical density, 0.212; process diameter, 10.6; maximum fragmentation, 4.38; activation threshold, 2.0). Briefly, annotation layers were created in accordance with Fig. 2A,B, and each region of interest was tuned with the set parameters to ensure accurate detection across the brain section.

### Statistical analysis

Data were stored in a MySQL database (v8.0.29) and graphed using GraphPad Prism (v9.5.0; GraphPad Software Inc., La Jolla, CA). Because of the small sample sizes, statistical analyses were not performed. However, we report all data to help inform future studies. All data points are an average for each animal, with the median and range for each experimental group presented.

## Results

Reported median CHIMERA velocity in the 22J group was 14.76 [14.44–15.10] m/s and in the 27J group velocity was 16.99 [16.44–17.00] m/s. All surviving animals recovered quickly and returned to normal behavior within 30 min after the TBI/sham procedure. We observed minimal changes in body weight across the 24 h period for shams (+10 g [–10 to +60]), 22J (5 g [–20 to +40]), and 27J (0 g [–30 to +5]).

### Physiological parameters

Heart rate decreased over the course of the procedure for the sham group, whereas in the injured animals heart rate was either maintained (22J) or elevated (27J) post-TBI (Table 1). Blood pressure was increased 5 min post-TBI in the 22J group. Unfortunately, no values were recorded in the 27J group immediately post-TBI because the animals required assistance to maintain their airway attributable to post-TBI bleeding. Oxygen saturation remained within normal limits for all animals. Sham animals had a decreased respiratory rate compared to injured animals. This may explain the elevated end-tidal CO_2_ for this group. The 22J group saw a brief increase in end-tidal CO_2_, whereas the 27J group saw little change in end-tidal CO_2_.

**Table 1.** Summary of All Non-Invasive Physiological Parameters in the First 15 min Post-TBI

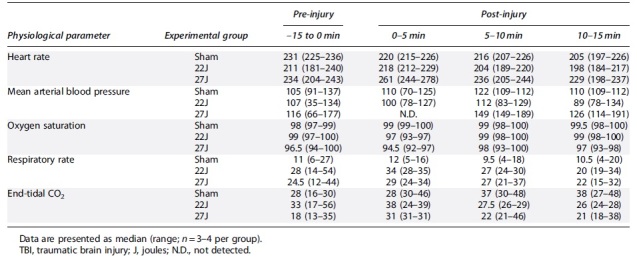

### Gross neuropathology

**FIG. 3.**
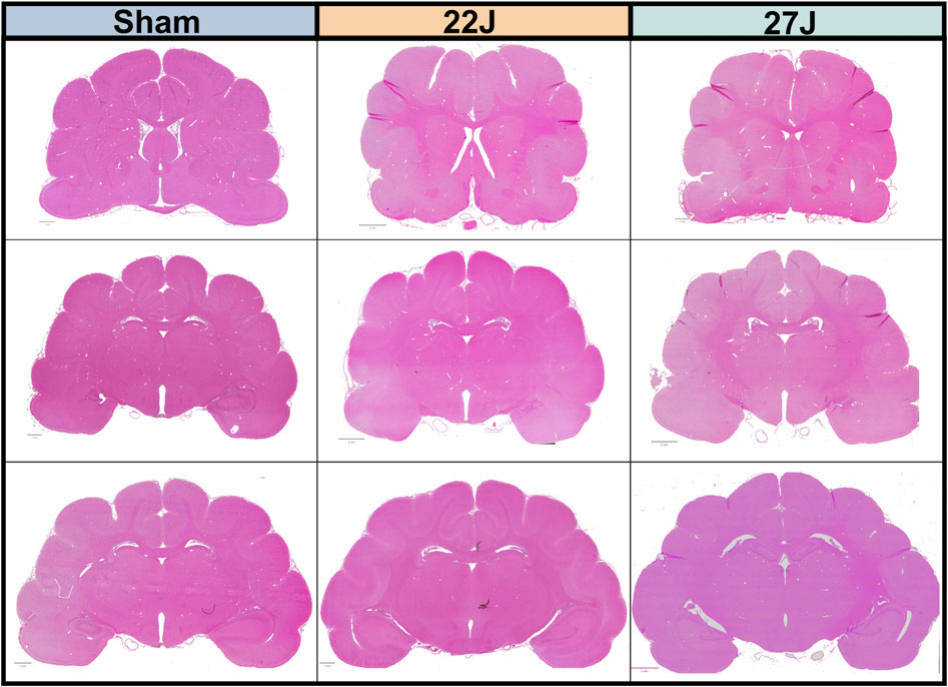
Representative H&E sections from all cerebrum anteroposterior levels and experimental groups. This shows no discernible lesions associated with this injury model at the levels assessed. H&E, hematoxylin and eosin.

Unfortunately, 1 animal died in the 27J group immediately after injury because of extensive skull fracturing and associated hemorrhage. This fracture is the equivalent of an anterior transverse fracture of the basilar skull, typically only observed in severe TBI.^34^ This animal was excluded from any analysis. Two of the remaining 3 animals had skull fracturing—one being a unilateral simple linear fracture running anterior to posterior and the other similar, but bilateral. No fractures were observed in the 22J group. These same 2 animals also had minor bleeding from the nasal cavity, which was controlled with gauze. Post-injury, the brains of all animals were normal on gross pathological examination and indistinguishable from sham animals. No evidence of focal lesions, including contusions, was noted. In line with these observations, H&E staining showed no hemorrhagic or ischemic lesions (Fig. 3).

### Immunohistochemistry

#### Amyloid precursor protein

**FIG. 4.**
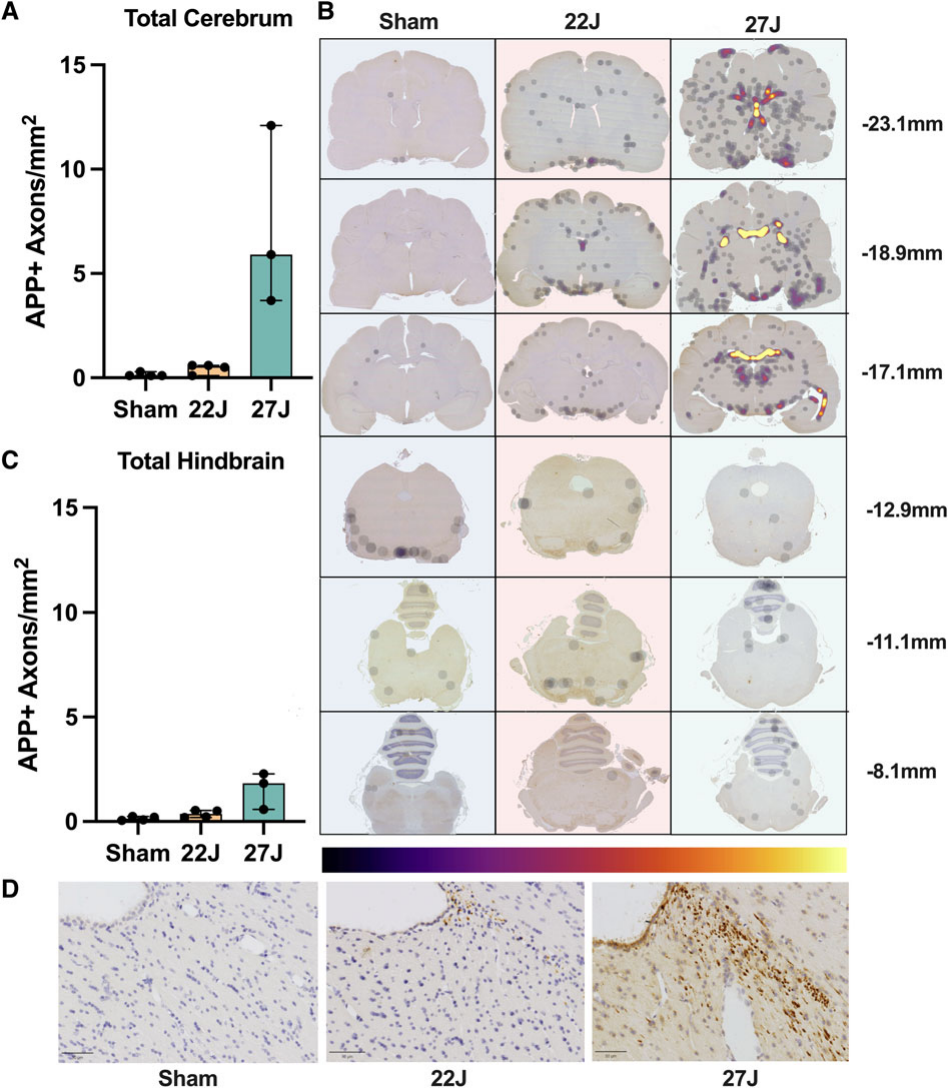
(**A**) Summary values of full cerebrum count of APP^+^ axons. This shows a step-wise increase in pathology with injury severity. (**B**) Representative density maps show each experimental group and approximate anteroposterior level. This highlights where axonal pathology is concentrated, with each black dot presenting an individual injured axon; as the color changes with the color bar, it indicates an increase in detection density. This highlights a pattern of high-density paraventricular staining in the 27J group cerebrum. No discernible patterns were observed in brainstem sections. (**C**) Total APP within the hindbrain. (**D**) Representative APP staining of the fornix/corpus callosum. Scale bar = 50 μm. Data are expressed as median ± range (*n* = 3–4). APP, amyloid precursor protein.

Total APP immunoreactivity across the entire stained area showed a 7.62-fold increase in the 22J TBI animals and a 74.25-fold increase in the 27J group (sham: 0.08 [0.08–0.302]; 22J: 0.53 [0.11–0.63]; 27J: 5.86 [3.69–12.10] APP^+^ axons/mm^2^; Fig. 4A). Spatial distribution on representative density maps (Fig. 4B) demonstrated a concentration of APP-positive axons on the ventral surface of the cerebrum in both the 22 and 27J groups and a marked increase in midline structures that interface with the lateral ventricles in the 27J group. All regions of interest with APP-positive axons are summarized in Table 2, with the full data set in the Supplementary Data. The fornix showed a substantial increase in APP pathology (22J: 21.00-fold; 27J: 2881-fold), with the cingulum and corpus callosum also showing notable APP immunoreactivity, and, to a lesser extent, the cortical white matter and internal capsule. Of the gray matter structures examined, the hypothalamus (22J: 10.32-fold; 27J: 54.95-fold) and thalamus (22J: 3.95-fold; 27J: 115.52-fold) showed the greatest amount of APP immunoreactivity, with the hippocampus and caudate showing smaller increases. APP immunoreactivity was much lower in the hindbrain (22J: 3.5-fold; 27J: 18.54-fold).

**Table 2.** Median and Range of APP-Positive Axons per mm^2^ for Each Anatomical Region of Interest

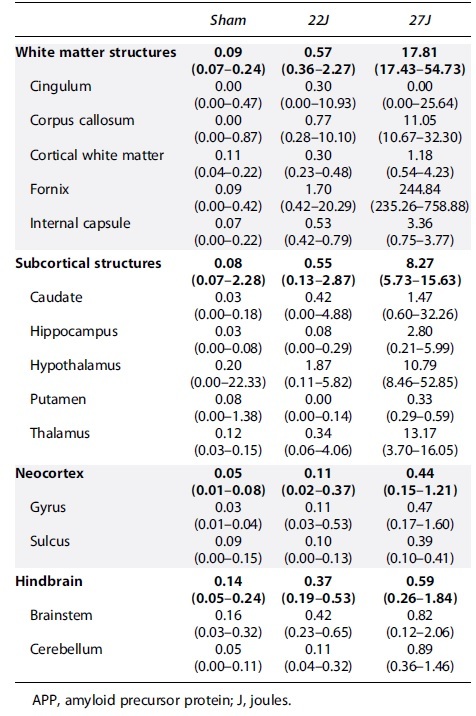

#### Neurofilament M (RMO 14.9)

**FIG. 5.**
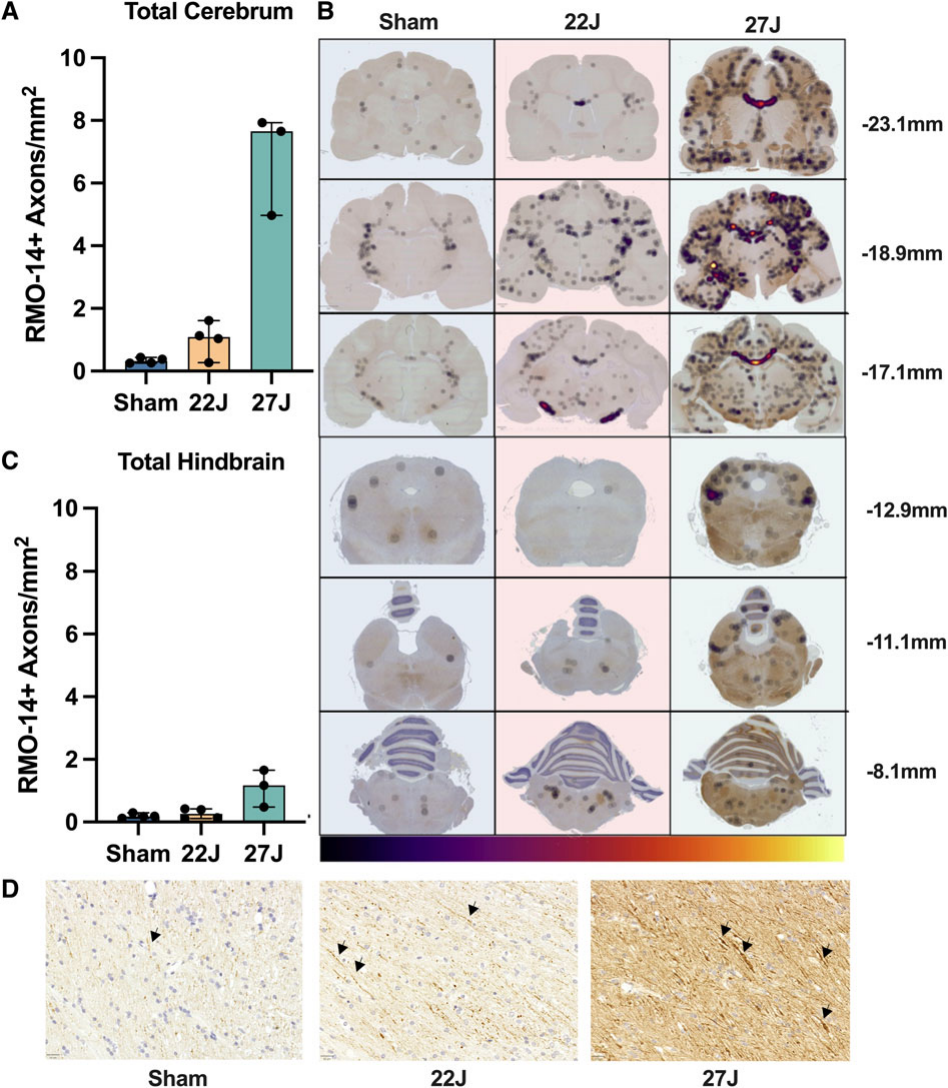
(**A**) Total cerebrum counts of median RMO-14^+^ axons per mm^2^. (**B**) Representative density maps of RMO-14^+^ axons, with the color bar indicating density. A step-wise increase with injury severity was observed in total brain counts. (**C**) Total median hindbrain RMO-14^+^ axons per mm^2^. (**D**) Representative RMO-14 staining of cortical white matter with arrows indicating axons that are RMO-14 positive based on staining intensity and morphology. Scale bar = 20 μm. Data are expressed as median ± range (*n* = 3–4). RMO-14, neurofilament M (RMO 14.9).

Total RMO-14 immunoreactivity across the cerebrum showed a 1.09-fold increase in the 22J group (1.09 [0.25–1.61] RMO-14^+^ axons/mm^2^) and a 7.65-fold increase in the 27J group (7.65 [4.97–7.93] RMO-14^+^ axons/mm^2^] compared against sham animals (0.32 [0.25–0.44] RMO-14^+^ axons/mm^2^; Fig. 5A). Representative density maps show a diffuse pattern of staining, with particularly strong staining within the corpus callosum of the 27J group (Fig. 5B). Within white matter, the strongest immunoreactivity compared to sham animals was within the corpus callosum (22J: 3.6-fold; 27J: 22.57-fold), with much smaller increases in the fornix, internal capsule, cerebral white matter, and cingulum (Table 3). Indeed, the lower 22J injury was insufficient to cause increases in RMO-14 immunoreactivity within the fornix and cingulum. Within subcortical structures, increases in RMO-14 pathology after the 27J impact were noted most prominently within the thalamus, hippocampus, and hypothalamus, with smaller increases in the caudate and putamen (Table 3). Negligible increases in RMO-14 immunoreactivity were noted with the 22J impact, except within the thalamus. Like the APP staining, the hindbrain showed much less RMO-14 immunoreactivity (22J: 2.45-fold; 27J: 7.44-fold), with only the brainstem in the 27J group showing increases in staining post-injury.

**Table 3.** Median and Range of RMO-14^+^ Axons per mm^2^ per Each Anatomical Region of Interest

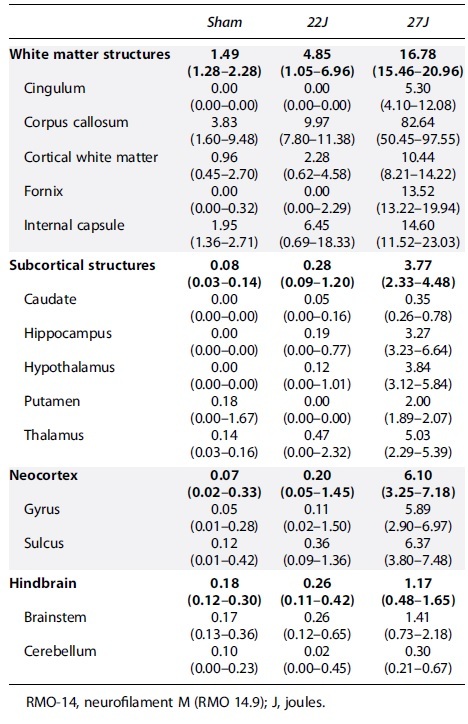

#### Analysis of the microglial response to injury

**FIG. 6.**
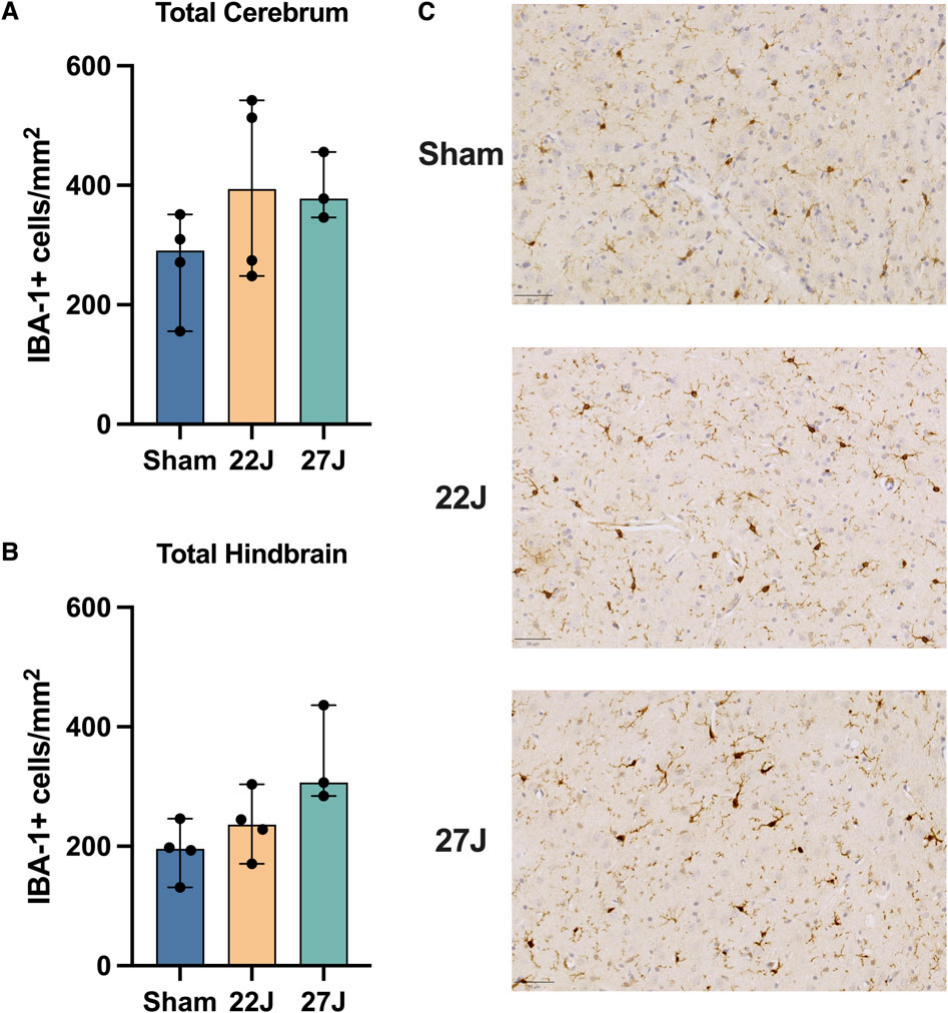
(**A**) Full cerebrum counts of median IBA-1^+^ cells per mm^2^ across the entire brain. (**B**) Total IBA-1^+^ cells per mm^2^ in the hindbrain. Both graphs show increased total microglia with injury. (**C**) Representative neocortex staining for IBA-1 across injury severity. Scale bar = 50 μm. Data are expressed as median ± range (*n* = 3–4). IBA-1, ionized calcium-binding adaptor molecule 1.

Full cerebrum counts of IBA-1^+^ cells/mm^2^ (Fig. 6A) revealed an increase in the density of IBA-1^+^ cells with injury (sham: 290.67 [158.03–351.08]; 22J: 393.73 [248.23–542.29]; 27J: 377.80 [346.36–455.85] IBA-1^+^ cells/mm^2^). A similar pattern was observed in the hindbrain (sham: 195.61 [131.19–246.43]; 22J: 251.52 [170.93–303.52]; 27J: 306.84 [284.29–436.10] IBA-1^+^ cells/mm^2^; Fig. 6B). Within the cerebrum, white matter structures saw the most prominent increases in the 27J group compared to sham animals (748.02 [527.87–767.62] vs. 206.94 [171.10–262.36] IBA-1^+^ cells/mm^2^), with a more modest increase in the 22J group (295.26 [256.32–334.34]). Of the white matter structures examined, the 27J impact caused the most substantial increase in the fornix (1020.40 [945.67–1282.36]) IBA-1^+^ cells/mm^2^, but all white matter structures showed a graded increase in IBA-1^+^ cell density depending on impact severity (Table 4). More modest increases were noted in subcortical structures, with a graded increase in IBA-1^+^ cells in the caudate, putamen, thalamus, and hypothalamus with increasing injury severity, but a decrease in the hippocampus (Table 4). The hindbrain (Fig. 6B) showed changes in microglia counts, increasing from 195.61 [131.19–246.43] in the sham group, 251.52 [170.93–303.52] in the 22J group, and 306.87 [284.29–436.10] in the 27J group.

**Table 4.** IBA-1-Positive Cells per mm^2^ in Each Region of Interest

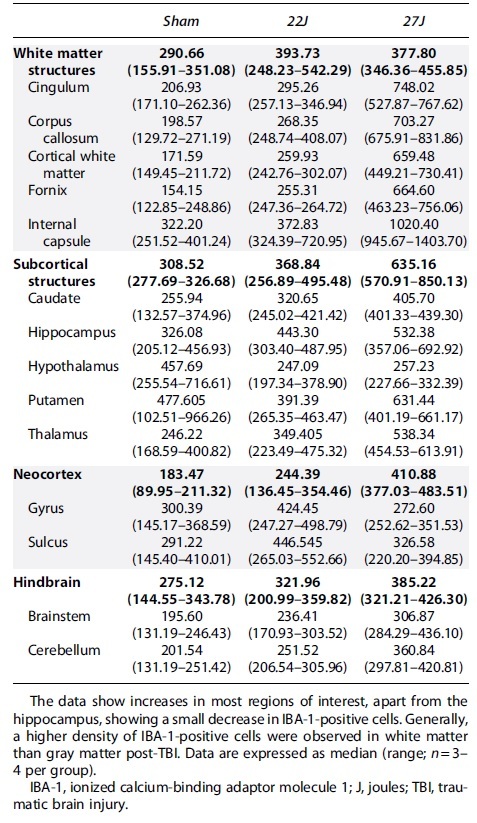

Microglial activation increased slightly with injury across all regions of interest Table 5 with the 22J group increasing between 1.22-fold in the hippocampus to 2.67-fold in the caudate, whereas the 27J group increased from 1.85-fold in the cerebellum to 3.73-fold in the cerebral cortex gyri.

**Table 5.** Microglial Activation Percentages According to the Indica Labs HALO Platform

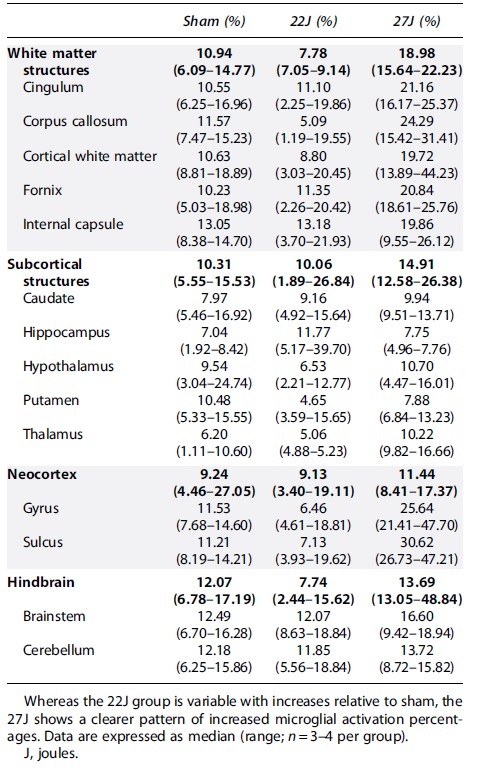

#### Phospho-tau

**FIG. 7.**
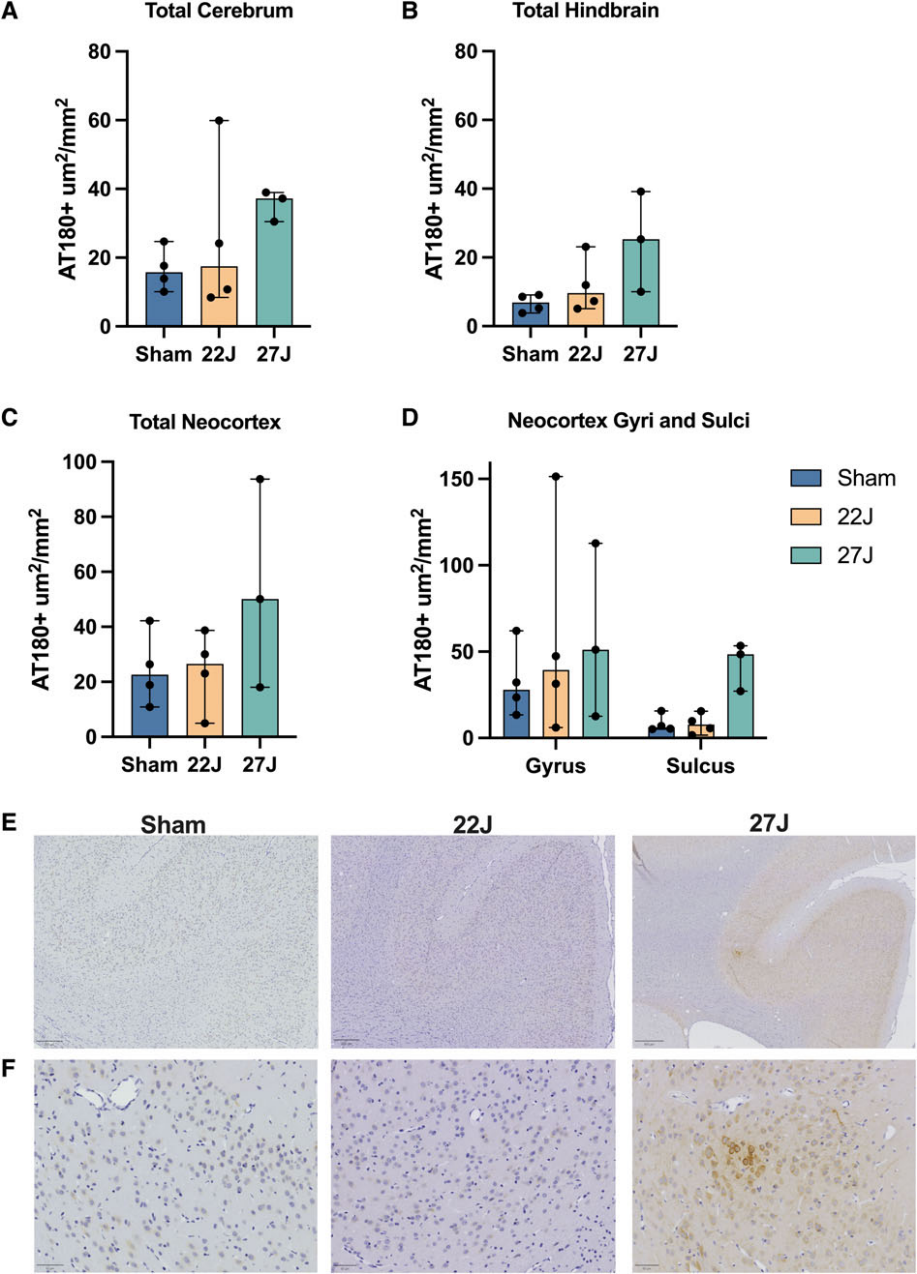
Total cerebrum AT-180^+^ staining in the cerebrum (**A**), hindbrain (**B**), neocortex (**C**), and gyri and sulci (**D**). A clear increase in phospho-tau staining in the sulcus of the 27J group, with no discernible changes in the gyri observed. (**E**) Representative AT180 staining of the splenial sulcus. Scale bar = 250 μm. (**F**) High-powered representative images of the same sulcus. Scale bar = 50 μm. Data are expressed as median ± range (*n* = 3–4).

The pattern of AT180 staining within the cortex was examined post-injury (Fig. 7). Evaluation of the neocortex found minimal differences with injury, with variability across the cohort (Fig. 7C). However, regional investigation found substantial increases in AT180-positive staining at the base of the sulci in the 27J group (48.42 [27.11–53.45] AT180^+^ μm^2^/mm^2^) compared to sham (6.22 [5.12–15.73] AT180^+^ μm^2^/mm^2^) or the 22J group (7.74 [1.61–15.51] AT180^+^ μm^2^/mm^2^; Fig. 7D). Hindbrain AT180 staining showed a graded increase from 7.77 [3.81–9.10] in the sham group, 10.92 [5.14–23.08] in the 22J group, and for the 27J group 32.54 [10.01–39.18] AT180^+^ μm^2^/mm^2^ (Fig. 7B), with this staining more prominent in the brainstem than the cerebellum. All regions of interest are presented in Table 6.

**Table 6.** Median and Range of AT180-Positive μm^2^ per mm^2^ per each Anatomical Region of Interest

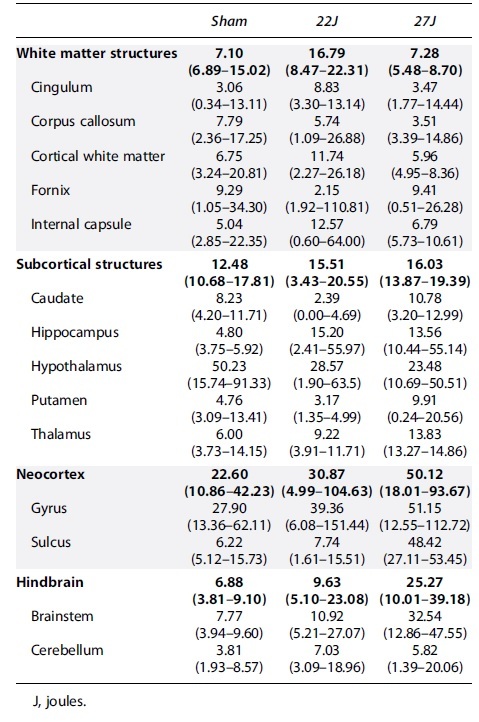

## Discussion

This study aimed to characterize the acute axonal injury and microglial response to TBI induced with the novel CHIMERA injury device in ferrets. The 22J group showed modest increases in axonal pathology, with no clear associated microglial activation. The 27J group showed pronounced DAI and increases in activated microglia, predominantly within the white matter. The study also used a wide scope of regions of interest to characterize the injury response spatially. Phosphorylated tau increased with injury severity, but only the 27J group showed a clear trend in sulci and brainstem tau accumulation.

The CHIMERA device has previously been used In mice showing graded diffuse axonal pathology, without focal pathology.^28,29^ Here, we similarly demonstrate the ability to produce graded axonal injury after a single impact in ferrets without focal injury, with a modest increase in axonal pathology observed with a 22J impact and a more substantial increase with a 27J impact. This increase was attributable to a 22% increase in impact energy. Similarly in a mouse CHIMERA study, increasing injury severity by 23% (1.7–2.1 J) led to a graded increase in pathology similar to what is reported here and mortality rates in the high-severity group (23% in mice; 25% in ferrets).^29^ Skull fracture incidence was lower in their high-severity group (18% in mice vs. 75% in ferrets), but this may be attributable to the use of an interface that dissipates force across the skull, which was not used in the present study.^29^ Future studies could incorporate a similar interface to investigate how this alters pathology.

DAI is best described as lesions in multiple yet common loci, particularly midline structures such as the corpus callosum, and increases with injury severity. Here, the 22J impact appears more akin to a mild injury and the 27J impact a more moderate injury based on the pattern of axonal injury. It should be noted that the mild classification has been challenged recently given that a proportion of patients remain symptomatic chronically.^3^ Although less prominent than the more severe 27J injury, axonal injury was evident in the 22J group in key white matter tracts like the corpus callosum, fornix, and cingulum. These axonal injury findings are in line with clinical post-mortem studies at more chronic time points demonstrating axonal injury in these regions even after mild TBI,^35^ with the extent of axonal injury increasing with injury severity.^36^

The amount of axonal injury markedly increased with the 27J impact, with prominent involvement of key subcortical structures and white matter tracts within the cerebrum and, to a lesser extent, the hindbrain, particularly the brainstem. Clinically, brainstem pathology is currently the diagnostic criterion for the highest DAI (grade 3), indicative of the observation of this pathology only in the 27J group here. Surprisingly, these results contradict preliminary neuroimaging investigation of the ferret CHIMERA model, which found altered fractional anisotropy in the brainstem, but not cerebrum, from 3 h post-injury. T2-weighted imaging was also suggestive of microhemorrhages and edema in the brainstem. However, the injury parameters differ; all ferrets in the Hutchinson and colleagues study were subject to different injury parameters with single or multiple impacts ranging from 15 to 75 PSI, making comparison difficult. Further, it was noted that the quality of diffusion magnetic resonance imaging maps was limited by short acquisition time, with future studies needed to assess the relationship between diffusion tensor imaging and axonal injury markers in the cerebrum and brainstem using consistent injury parameters.^31^

Nonetheless, axonal injury within white matter tracts and the brainstem was accompanied by a graded increase in IBA-1^+^ cells. Diffuse TBI is associated with accumulation of monocytes/macrophages within white matter tracts, which colocalize with axonal pathology.^37–39^ Clinically, post-mortem severe TBI studies have found higher numbers of microglia in white compared to gray matter, with increased microglial burden observed in brains where DAI is present.^40^ The increase in microglia number may reflect migration and/or proliferation of local microglia or infiltration of monocyte-derived macrophages.^41^ Results here differ from those reported in the single murine CHIMERA model, where increases in IBA-1^+^ cells were only noted from 2 days in white matter tracts after mild injury^28^ and an increase in IBA-1^+^ staining from 14 days in the moderate version of the injury.^27^ This may reflect differences in injury severity given the greater axonal burden noted here. Other differences could be attributable to interspecies differences in the microglia response. Finally, differences in the assessment of IBA-1 staining, with the moderate injury study only assessing the percentage area staining, not number of cells/mm^2^, may also account for this discrepancy.^27^

Notably, only the 27J group, but not the 22J injury, showed evidence of microglial activation, as defined by increased process thickness and reduced branch length as a measure of activation within these white matter tracts. Evidence of microglial activation in response to neuronal injury has been shown within 15 min in pig models of diffuse injury, which was graded in response to injury severity.^42^ Milder injury may lead to different phenotypic changes in microglia, such as an increase in process length and branching,^43,44^ which were not assessed with the HALO module utilized here. For example, in a mild head acceleration model of diffuse injury in pigs, alterations in microglia were detected as increases in the number of microglial branches, junctions, and end-points, rather than the decrease examined here.^45^ Further, morphological appearance is not sufficient to determine the function of microglia. More detailed analysis will be needed to examine the transcriptomic and proteomic responses within these microglia in response to injury.^46^

Of the gray matter structures, the thalamus, hypothalamus, and hippocampus showed the greatest degree of axonal injury. Given that the thalamus and hypothalamus are midline structures, have a fluid/tissue interface, and are in plane with the injury, it is likely that these axons have been exposed to a high degree of force and may be particularly vulnerable to this injury. This is supported in other diffuse TBI models, with the hypothalamus,^47,48^ thalamus,^38,49,50^ and hippocampus^51,52^ all having been shown to be vulnerable. This axonal injury was accompanied by a graded increase in microglia number in the thalamus and hippocampus, but not the hypothalamus. Nonetheless, no changes in activation status were found in either injury group within any of these gray matter structures, which may relate to the limitations of the analysis, which do not account for different microglial phenotypes dependent on injury severity, alongside the use of morphology to infer functionality of microglia. Further, microglia are known to converge specifically on damaged axons^53^ and neurons^42^ within the gray matter, and thus averaging the entire region may not be sensitive enough to detect regional alterations in microglia activation status. Future studies could utilize more targeted analysis and investigate the relationship specifically between microglial processes and damaged neurons and axons.

Axonal injury was evaluated by two markers, APP for impaired axonal transport and RMO-14 for neurofilament compaction, given that axons are known to show different pathology after TBI.^54^ However, previous reports have suggested that impaired axonal transport and neurofilament compaction in diffuse rodent models have the same anatomical distribution, even though they are often present in different axons.^55,56^ This is only partially supported here, with neurofilament compaction showing a more diffuse pattern of injury than that observed by APP. Interestingly, immunohistochemistry of fatal human severe TBI showed minimal overlap between neurofilament and APP staining within individual injured axons, but the broader anatomical distribution has not been examined.^54^ Here, we found that the markers were prominent in different locations; for APP this was the fornix, and for RMO-14 the corpus callosum.

The fornix and, to a lesser extent, the cingulum may be particularly vulnerable to transport disruption detected by APP given the high strains associated with their periventricular location,^45,52^ the transverse directionality of their fibers increasing vulnerability to stretching, and their smaller caliber increasing the microtubule-to-neurofilament ratio. Indeed, APP immunoreactivity was particularly concentrated in periventricular areas, in line with other gyrencephalic pig studies of DAI^45,57^ and clinical studies, were fatal falls are associated with increased periventricular DAI, and strong staining in the fornix and corpus callosum.^11^ Indeed, finite element computer modeling using a human brain with and without ventricles suggests that the presence of ventricles increased strain in these periventricular regions, as supported here.^57^ Alongside the fornix as the key output for the hippocampus, the periventricular white matter tracts contain long association tracts governing more distant cortical areas and may play an important role in our cognitive functions, which needs to be investigated in future studies.

As observed by the differing pattern of RMO-14 staining, axons that do not show transport disruption may still show pathophysiological changes. RMO-14 is a marker of neurofilament compaction,^58^ where interfilament spacing is reduced because of side-arm phosphorylation and proteolysis,^59^ partially in response to increased intra-axonal sodium and calcium-induced protease activation. Compaction allows access of RMO-14 to the central rod domains.^60^ Here, it appears that this neurofilament compaction preferentially occurred in the corpus callosum, and as such appears to be the result of direct deformation given the location directly under the impact site and the transverse direction of the fibers work.^30^ Future studies will need to investigate the propensity for different white fiber tracts to demonstrate differing axonal pathology post-injury and its temporal profile.

Axonal injury is also thought to be a key driver of tau pathology post-TBI. The strain placed on axons causes tau proteins to become stiffer, inhibiting microtubules from sliding past each other in response to stretching leading to rupture.^61^ As tau becomes unbound from the microtubule, it facilitates phosphorylation at disease-related sites^62–65^ and promotes aggregation into oligomers and neurofibrillary tangles.^66–68^ Aggregates of tau have been observed in the base of the sulci after a single moderate-to-severe TBI,^69^ in line with computational and physical modeling that sulci receive more strain compared to the rest of the neocortex.^14^ Only the 27J impact was sufficient to drive increases of AT180 immunoreactivity, which was only evident at the base of the sulci and not the surrounding gyri. In some cases, repeated TBIs have been associated with the later development of chronic traumatic encephalopathy (CTE), with the pathognomonic lesion hyperphosphorylated tau aggregates in neurons and astrocytes close to blood vessels at the base of the sulci.^70^ Whether a single, more severe impact is sufficient to drive CTE development is not known, with further studies needed to investigate the temporal profile of tau changes after a single injury in this model and the effects of repeated injury with the milder 22J impact.

The ferret brain offers several advantages given its similarity to the human brain. It has gyri and sulci,^22^ subcortical U fibers crucial for cognition,^71^ a ventrally situated hippocampus, and separation of the caudate and putamen by the internal capsule^18^—features not noted in rodents. Indeed, key clinical histopathological features of diffuse injury were able to be replicated with the CHIMERA model in the gyrencephalic ferret. In line with clinical reports, the milder 22J impact was associated with more discrete foci of axonal injury, particularly within periventricular areas including the fornix and corpus callosum. Clinically, axonal pathology detected within 6 months of mild TBI in patients who died from other causes similarly shows axonal injury detected by APP^35^ or neurofilament staining^72^ in the fornix, corpus callosum, and white matter of the cerebrum. Neuroimaging studies within 1 week of mild TBI have also found alterations in fractional anisotropy suggestive of axonal injury within the fornix and corpus callosum.^73^

With increasing injury severity in our model, axonal injury was found more extensively throughout white matter tracts of the cerebrum, subcortical structures, and within the hindbrain. In clinical TBI, more severe injury is similarly associated with more extensive axonal injury and inflammation with involvement of subcortical structures, including the thalamus, hypothalamus,^74^ and hippocampus,^75^ as noted here. The pattern of axonal injury observed clinically after diffuse injury, particularly in white matter tracts, is not reliably replicated by rodent models. For example, in the original Marmarou weight-drop model optimization, prominent axonal injury was reported within the brainstem, with less severe injury in the corpus callosum and internal capsule. Indeed, a more recent study incorporating the model similarly found prominent axonal injury in the corpus callosum and optic tract, but not the fornix.^43^ This is not injury-model specific, with moderate-to-severe CHIMERA TBI in the mouse associated with axonal pathology with accompanying inflammation in the optic tract, but not the corpus callosum, with no mention of the fornix.^27^

## Conclusion

Although diffuse injury models have been developed in sheep^76^ and pigs,^52,77^ this is the first article to characterize the histological response to a CHIMERA impact-induced single diffuse injury in a small gyrencephalic ferret model. Graded axonal and microglial pathology was observed with increasing injury severity, with differing patterns of axonal injury detected dependent on the marker used. APP-positive axonal injury indicative of axonal transport disruption was particularly prominent within the periventricular region, whereas RMO-14-positive axonal injury showing neurofilament compaction was more diffuse. Of note, the more severe injury was associated with accumulation of hyperphosphorylated tau at the base of the sulci, with further studies needed to investigate how this pathology evolves over time. Thus, we propose that this model has utility in exploring different axonal injury phenotypes and how this may evolve to chronic white matter degeneration in future studies.

## Supplementary Material

Supplemental data

Supplemental data

Supplemental data

Supplemental data

## Transparency, Rigor, and Reproducibility Summary

The analysis plan was not formally pre-registered, but the senior author with primary responsibility for the analysis certifies that the analysis plan was pre-specified. Twelve ferrets were included within the experiment as a proof of concept and randomly assigned to groups using a random number generator. All animals reached the assigned end-point of the study aside from the one animal euthanized because of a catastrophic skull fracture. Investigators who performed histological analyses were blinded to group by coding the slides. All materials used to conduct the study were obtained from a widely available source, as stated within the methods of the article. The authors agree to provide the full content of the manuscript on request by contacting the corresponding author. Samples collected as part of the study are available to be used for future research without requirement for specific additional informed consent or regulatory controls. Please contact the corresponding author.

## Abbreviations Used

APP: amyloid precursor protein
CHIMERA: Closed Head Injury Model of Engineered Rotational Acceleration (CHIMERA)
CTE: chronic traumatic encephalopathy
DAB: 3,3-diaminobenzidine
DAI: diffuse axonal injury
H&E: hemoxylin and eosin
IBA-1: ionized calcium-binding adaptor molecule 1
J: joules
NHS: normal horse serum
PSI: pounds per square inch
RMO-14: neurofilament M (RMO 14.9)
TBI: traumatic brain injury

## References

[1] Faul M, Wald MM, Xu L, et al. Traumatic brain injury in the United States: emergency department visits, hospitalizations, and deaths, 2002–2006. Centers for Disease Control and Prevention, National Center for Injury Prevention and Control: Atlanta, GA; 2010.

[2] Gardner RC, Yaffe K. Epidemiology of mild traumatic brain injury and neurodegenerative disease. Mol Cell Neurosci 2015;66(Pt B):75–80; doi: 10.1016/j.mcn.2015.03.001 25748121 PMC4461453

[3] Nelson LD, Temkin NR, Dikmen S, et al. Recovery after mild traumatic brain injury in patients presenting to US Level I trauma centers: a Transforming Research and Clinical Knowledge in Traumatic Brain Injury (TRACK-TBI) study. JAMA Neurol 2019;76(9):1049–1059; doi: 10.1001/jamaneurol.2019.1313 31157856 PMC6547159

[4] Dewan MC, Rattani A, Gupta S, et al. Estimating the global incidence of traumatic brain injury. J Neurosurg 2019;130(4):1080–1097; doi: 10.3171/2017.10.JNS17352 29701556

[5] McGinn MJ, Povlishock JT. Pathophysiology of traumatic brain injury. Neurosurg Clin N Am 2016;27(4):397–407; doi: 10.1016/j.nec.2016.06.002 27637392

[6] Chaban V, Clarke GJB, Skandsen T, et al. Systemic inflammation persists the first year after mild traumatic brain injury: results from the Prospective Trondheim Mild Traumatic Brain Injury Study. J Neurotrauma 2020;37(19):2120–2130; doi: 10.1089/neu.2019.6963 32326805 PMC7502683

[7] Kumar RG, Boles JA, Wagner AK. Chronic inflammation after severe traumatic brain injury: characterization and associations with outcome at 6 and 12 months postinjury. J Head Trauma Rehabil 2015;30(6):369–381; doi: 10.1097/HTR.0000000000000067 24901329

[8] Büki A, Povlishock JT. All roads lead to disconnection?—Traumatic axonal injury revisited. Acta Neurochir Wien 2006;148(2):181–193; discussion, 193; doi: 10.1007/s00701-005-0674-4 16362181

[9] Tang-Schomer MD, Patel AR, Baas PW, et al. Mechanical breaking of microtubules in axons during dynamic stretch injury underlies delayed elasticity, microtubule disassembly, and axon degeneration. FASEB J 2010;24(5):1401–1410; doi: 10.1096/fj.09-142844 20019243 PMC2879950

[10] Smith DH, Wolf JA, Lusardi TA, et al. High tolerance and delayed elastic response of cultured axons to dynamic stretch injury. J Neurosci 1999;19(11):4263–4269; doi: 10.1523/JNEUROSCI.19-11-04263.1999 10341230 PMC6782601

[11] Blumbergs PC, Scott G, Manavis J, et al. Topography of axonal injury as defined by amyloid precursor protein and the sector scoring method in mild and severe closed head injury. J Neurotrauma 1995;12(4):565–572; doi: 10.1089/neu.1995.12.565 8683607

[12] Bruggeman GF, Haitsma IK, Dirven CMF, et al. Traumatic axonal injury (TAI): definitions, pathophysiology and imaging-a narrative review. Acta Neurochir Wien 2021;163(1):31–44; doi: 10.1007/s00701-020-04594-1 33006648 PMC7778615

[13] Davceva N, Sivevski A, Basheska N. Traumatic axonal injury, a clinical-pathological correlation. J Forensic Leg Med 2017;48:35–40; doi: 10.1016/j.jflm.2017.04.004 28437717

[14] Cloots RJ, Gervaise HM, van Dommelen JA, et al. Biomechanics of traumatic brain injury: influences of the morphologic heterogeneities of the cerebral cortex. Ann Biomed Eng 2008;36(7):1203–1215; doi: 10.1007/s10439-008-9510-3 18465248 PMC2413127

[15] Sorby-Adams AJ, Vink R, Turner RJ. Large animal models of stroke and traumatic brain injury as translational tools. Am J Physiol-Regul Integr Comp Physiol 2018;315(2):R165–R190; doi: 10.1152/ajpregu.00163.2017 29537289

[16] Vink R. Large animal models of traumatic brain injury. J Neurosci Res 2018;96(4):527–535; doi: 10.1002/jnr.24079 28500771

[17] Duque A, McCormick DA. Circuit-based localization of ferret prefrontal cortex. Cereb Cortex 2010;20(5):1020–1036; doi: 10.1093/cercor/bhp164 19737780 PMC2852501

[18] Radtke-Schuller S. Cyto- and Myeloarchitectural Brain Atlas of the Ferret (Mustela Putorius) in MRI Aided Stereotaxic Coordinates. Springer International Publishing AG: Cham, Switzerland; 2018.

[19] Hutsler JJ, Lee DG, Porter KK. Comparative analysis of cortical layering and supragranular layer enlargement in rodent carnivore and primate species. Brain Res 2005;1052(1):71–81; doi: 10.1016/j.brainres.2005.06.015 16018988

[20] Lighthall JW. Controlled cortical impact: a new experimental brain injury model. J Neurotrauma 1988;5(1):1–15; doi: 10.1089/neu.1988.5.1 3193461

[21] Schwerin SC, Chatterjee M, Imam-Fulani AO, et al. Progression of histopathological and behavioral abnormalities following mild traumatic brain injury in the male ferret. J Neurosci Res 2018;96(4):556–572; doi: 10.1002/jnr.24218 29360208

[22] Schwerin SC, Hutchinson EB, Radomski KL, et al. Establishing the ferret as a gyrencephalic animal model of traumatic brain injury: optimization of controlled cortical impact procedures. J Neurosci Methods 2017;285:82–96; doi: 10.1016/.2017.05.010 28499842 PMC6320441

[23] Rafaels KA, Bass CR, Panzer MB, et al. Brain injury risk from primary blast. J Trauma Acute Care Surg 2012;73(4):895–901; doi: 10.1097/TA.0b013e31825a760e 22836001

[24] Schwerin SC, Chatterjee M, Hutchinson EB, et al. Expression of GFAP and tau following blast exposure in the cerebral cortex of ferrets. J Neuropathol Exp Neurol 2021;80(2):112–128; doi: 10.1093/jnen/nlaa157 33421075 PMC8453607

[25] Cernak I. The importance of systemic response in the pathobiology of blast-induced neurotrauma. Front Neurol 2010;1:151; doi: 10.3389/fneur.2010.00151 21206523 PMC3009449

[26] Namjoshi DR, Cheng WH, McInnes KA, et al. Merging pathology with biomechanics using CHIMERA (Closed-Head Impact Model of Engineered Rotational Acceleration): a novel, surgery-free model of traumatic brain injury. Mol Neurodegener 2014;9(1):55; doi: 10.1186/1750-1326-9-55 25443413 PMC4269957

[27] Bashir A, Abebe ZA, McInnes KA, et al. Increased severity of the CHIMERA model induces acute vascular injury, sub-acute deficits in memory recall, and chronic white matter gliosis. Exp Neurol 2020;324:113116; doi: 10.1016/j.expneurol.2019.113116 31734317

[28] Namjoshi DR, Cheng WH, Bashir A, et al. Defining the biomechanical and biological threshold of murine mild traumatic brain injury using CHIMERA (Closed Head Impact Model of Engineered Rotational Acceleration). Exp Neurol 2017;292:80–91; doi: 10.1016/j.expneurol.2017.03.003 28274861

[29] Sauerbeck AD, Fanizzi C, Kim JH, et al. modCHIMERA: a novel murine closed-head model of moderate traumatic brain injury. Sci Rep 2018;8(1):7677; doi: 10.1038/s41598-018-25737-6 29769541 PMC5955903

[30] Sun C, Qi L, Cheng Y, et al. Immediate induction of varicosities by transverse compression but not uniaxial stretch in axon mechanosensation. Acta Neuropathol Commun 2022;10(1):7; doi: 10.1186/s40478-022-01309-8 35074017 PMC8785443

[31] Hutchinson EB, Romero-Lozano A, Johnson HR, et al. Translationally relevant magnetic resonance imaging markers in a ferret model of closed head injury. Front Neurosci 2022;15:779533; doi: 10.3389/fnins.2021.779533 35280340 PMC8904401

[32] National Health and Medical Research Council (Australia). Australian Code for the Care and Use of Animals for Scientific Purposes. National Health and Medical Research Council: Canberra; 2013.

[33] Bankhead P, Loughrey MB, Fernández JA, et al. QuPath: open source software for digital pathology image analysis. Sci Rep 2017;7(1):16878; doi: 10.1038/s41598-017-17204-5 29203879 PMC5715110

[34] Simon LV, Newton EJ. Basilar Skull Fractures. StatPearls Publishing: Treasure Island, FL; 2022. 29261908

[35] Blumbergs PC, Scott G, Manavis J, et al. Stalning af amyloid percursor protein to study axonal damage in mild head injury. Lancet 1994;344(8929):1055–1056; doi: 10.1016/S0140-6736(94)91712-4 7523810

[36] Johnson VE, Stewart JE, Begbie FD, et al. Inflammation and white matter degeneration persist for years after a single traumatic brain injury. Brain 2013;136(Pt 1):28–42; doi: 10.1093/brain/aws322 23365092 PMC3562078

[37] Hellewell SC, Yan EB, Agyapomaa DA, et al. Post-traumatic hypoxia exacerbates brain tissue damage: analysis of axonal injury and glial responses. J Neurotrauma 2010;27(11):1997–2010; doi: 10.1089/neu.2009.1245 20822466

[38] Kelley BJ, Lifshitz J, Povlishock JT. Neuroinflammatory responses after experimental diffuse traumatic brain injury. J Neuropathol Exp Neurol 2007;66(11):989–1001; doi: 10.1097/NEN.0b013e3181588245 17984681

[39] Oehmichen M, Theuerkauf I, Meissner C. Is traumatic axonal injury (AI) associated with an early microglial activation? Application of a double-labeling technique for simultaneous detection of microglia and AI. Acta Neuropathol (Berl) 1999;97(5):491–494; doi: 10.1007/s004010051018 10334486

[40] Smith C, Gentleman SM, Leclercq PD, et al. The neuroinflammatory response in humans after traumatic brain injury. Neuropathol Appl Neurobiol 2013;39(6):654–666; doi: 10.1111/nan.12008 23231074 PMC3833642

[41] Spiteri AG, Wishart CL, Pamphlett R, et al. Microglia and monocytes in inflammatory CNS disease: integrating phenotype and function. Acta Neuropathol (Berl) 2022;143(2):179–224; doi: 10.1007/s00401-021-02384-2 34853891 PMC8742818

[42] Wofford KL, Harris JP, Browne KD, et al. Rapid neuroinflammatory response localized to injured neurons after diffuse traumatic brain injury in swine. Exp Neurol 2017;290:85–94; doi: 10.1016/j.expneurol.2017.01.004 28081963 PMC5529036

[43] Mohamed AZ, Corrigan F, Collins-Praino LE, et al. Evaluating spatiotemporal microstructural alterations following diffuse traumatic brain injury. Neuroimage Clin 2020;25:102136; doi: 10.1016/j.nicl.2019.102136 31865019 PMC6931220

[44] Morrison H, Young K, Qureshi M, et al. Quantitative microglia analyses reveal diverse morphologic responses in the rat cortex after diffuse brain injury. Sci Rep 2017;7:13211; doi: 10.1038/s41598-017-13581-z 29038483 PMC5643511

[45] Grovola MR, Paleologos N, Brown DP, et al. Diverse changes in microglia morphology and axonal pathology during the course of 1 year after mild traumatic brain injury in pigs. Brain Pathol 2021;31(5):e12953; doi: 10.1111/bpa.12953 33960556 PMC8412066

[46] Wang J, He W, Zhang J. A richer and more diverse future for microglia phenotypes. Heliyon 2023;9(4):e14713; doi: 10.1016/j.heliyon.2023.e14713 37025898 PMC10070543

[47] Bromberg CE, Condon AM, Ridgway SW, et al. Sex-dependent pathology in the HPA axis at a sub-acute period after experimental traumatic brain injury. Front Neurol 2020;11:946; doi: 10.3389/fneur.2020.00946 33101162 PMC7554641

[48] Valko PO, Gavrilov YV, Yamamoto M, et al. Damage to arousal-promoting brainstem neurons with traumatic brain injury. Sleep 2016;39(6):1249–1252; doi: 10.5665/sleep.5844 27091531 PMC4863213

[49] Grossman EJ, Inglese M. The role of thalamic damage in mild traumatic brain injury. J Neurotrauma 2016;33(2):163–167; doi: 10.1089/neu.2015.3965 26054745 PMC4722574

[50] Thomas TC, Ogle SB, Rumney BM, et al. Does time heal all wounds? Experimental diffuse traumatic brain injury results in persisting histopathology in the thalamus. Behav Brain Res 2018;340:137–146; doi: 10.1016/j.bbr.2016.12.038 28042008 PMC5491365

[51] Carron SF, Yan EB, Allitt BJ, et al. Immediate and medium-term changes in cortical and hippocampal inhibitory neuronal populations after diffuse TBI. Neuroscience 2018;388:152–170; doi: 10.1016/j.neuroscience.2018.07.020 30036662

[52] Smith DH, Chen XH, Xu BN, et al. Characterization of diffuse axonal pathology and selective hippocampal damage following inertial brain trauma in the pig. J Neuropathol Exp Neurol 1997;56(7):822–834; doi: 10.1097/00005072-199756070-00009 9210879

[53] Lafrenaye AD, Todani M, Walker SA, et al. Microglia processes associate with diffusely injured axons following mild traumatic brain injury in the micro pig. J Neuroinflammation 2015;12:186; doi: 10.1186/s12974-015-0405-6 26438203 PMC4595283

[54] Johnson VE, Stewart W, Weber MT, et al. SNTF immunostaining reveals previously undetected axonal pathology in traumatic brain injury. Acta Neuropathol 2016;131(1):115–135; doi: 10.1007/s00401-015-1506-0 26589592 PMC4780426

[55] Creed JA, DiLeonardi AM, Fox DP, et al. Concussive brain trauma in the mouse results in acute cognitive deficits and sustained impairment of axonal function. J Neurotrauma 2011;28(4):547–563; doi: 10.1089/neu.2010.1729 21299360 PMC3070143

[56] DiLeonardi AM, Huh JW, Raghupathi R. Impaired axonal transport and neurofilament compaction occur in I populations of injured axons following diffuse brain injury in the immature rat. Brain Res 2009;1263:174–182; doi: 10.1016/j.brainres.2009.01.021 19368848 PMC2696174

[57] Song H, McEwan PP, Ameen-Ali KE, et al. Concussion leads to widespread axonal sodium channel loss and disruption of the node of Ranvier. Acta Neuropathol (Berl) 2022;144(5):967–985; doi: 10.1007/s00401-022-02498-1 36107227 PMC9547928

[58] Marmarou CR, Walker SA, Davis CL, et al. Quantitative analysis of the relationship between intra- axonal neurofilament compaction and impaired axonal transport following diffuse traumatic brain injury. J Neurotrauma 2005;22(10):1066–1080; doi: 10.1089/neu.2005.22.1066 16238484

[59] Yuan A, Rao MV, Veeranna, et al. Neurofilaments and neurofilament proteins in health and disease. Cold Spring Harb Perspect Biol 2017;9(4):a018309; doi: 10.1101/cshperspect.a018309 28373358 PMC5378049

[60] Hall GF, Lee VM. Neurofilament sidearm proteolysis is a prominent early effect of axotomy in lamprey giant central neurons. J Comp Neurol 1995;353(1):38–49; doi: 10.1002/cne.903530106 7714248

[61] Ahmadzadeh H, Smith DH, Shenoy VB. Mechanical effects of dynamic binding between tau proteins on microtubules during axonal injury. Biophys J 2015;109(11):2328–2337; doi: 10.1016/j.bpj.2015.09.010 26636944 PMC4675823

[62] Ballatore C, Lee VM, Trojanowski JQ. Tau-mediated neurodegeneration in Alzheimer's disease and related disorders. Nat Rev Neurosci 2007;8(9):663–672; doi: 10.1038/nrn2194 17684513

[63] Planel E, Krishnamurthy P, Miyasaka T, et al. Anesthesia-induced hyperphosphorylation detaches 3-repeat tau from microtubules without affecting their stability in vivo. J Neurosci 2008;28(48):12798–12807; doi: 10.1523/JNEUROSCI.4101-08.2008 19036972 PMC2610528

[64] Feuillette S, Miguel L, Frébourg T, et al. Drosophila models of human tauopathies indicate that Tau protein toxicity in vivo is mediated by soluble cytosolic phosphorylated forms of the protein. J Neurochem 2010;113(4):895–903; doi: 10.1111/j.1471-4159.2010.06663.x 20193038

[65] Miyasaka T, Sato S, Tatebayashi Y, et al. Microtubule destruction induces tau liberation and its subsequent phosphorylation. FEBS Lett 2010;584(14):3227–3232; doi: 10.1016/j.febslet.2010.06.014 20561519

[66] Perez M, Santa-María I, Tortosa E, et al. The role of the VQIVYK peptide in tau protein phosphorylation. J Neurochem 2007;103(4):1447–1460; doi: 10.1111/j.1471-4159.2007.04834.x 17680993

[67] Thies E, Mandelkow E-M. Missorting of tau in neurons causes degeneration of synapses that can be rescued by the kinase MARK2/Par-1. J Neurosci 2007;27(11):2896–2907; doi: 10.1523/JNEUROSCI.4674-06.2007 17360912 PMC6672561

[68] Fischer D, Mukrasch MD, Biernat J, et al. Conformational changes specific for pseudophosphorylation at serine 262 selectively impair binding of tau to microtubules. Biochemistry 2009;48(42):10047–10055; doi: 10.1021/bi901090m 19769346

[69] Johnson VE, Stewart W, Smith DH. Widespread tau and amyloid-beta pathology many years after a single traumatic brain injury in humans. Brain Pathol 2012;22(2):142–149; doi: 10.1111/j.1750-3639.2011.00513.x 21714827 PMC3979351

[70] Murray HC, Osterman C, Bell P, et al. Neuropathology in chronic traumatic encephalopathy: a systematic review of comparative post-mortem histology literature. Acta Neuropathol Commun 2022;10(1):108; doi: 10.1186/s40478-022-01413-9 35933388 PMC9356428

[71] Yoshino M, Saito K, Kawasaki K, et al. The origin and development of subcortical U-fibers in gyrencephalic ferrets. Mol Brain 2020;13(1):37; doi: 10.1186/s13041-020-00575-8 32156301 PMC7063767

[72] McKee AC, Daneshvar DH, Alvarez VE, et al. The neuropathology of sport. Acta Neuropathol (Berl) 2014;127(1):29–51; doi: 10.1007/s00401-013-1230-6 24366527 PMC4255282

[73] Inglese M, Makani S, Johnson G, et al. Diffuse axonal injury in mild traumatic brain injury: a diffusion tensor imaging study. J Neurosurg 2005;103(2):298–303; doi: 10.3171/jns.2005.103.2.0298 16175860

[74] Valko PO, Gavrilov YV, Yamamoto M, et al. Damage to histaminergic tuberomammillary neurons and other hypothalamic neurons with traumatic brain injury. Ann Neurol 2015;77(1):177–182; doi: 10.1002/ana.24298 25363332

[75] Kotapka MJ, Graham DI, Adams JH, et al. Hippocampal pathology in fatal human head injury without high intracranial pressure. J Neurotrauma 1994;11(3):317–324; doi: 10.1089/neu.1994.11.317 7996585

[76] Anderson RWG, Brown CJ, Blumbergs PC, et al. Impact mechanics and axonal injury in a sheep model. J Neurotrauma 2003;20(10):961–974; doi: 10.1089/089771503770195812 14588113

[77] Browne KD, Chen XH, Meaney DF, et al. Mild traumatic brain injury and diffuse axonal injury in swine. J Neurotrauma 2011;28(9):1747–1755; doi: 10.1089/neu.2011.1913 21740133 PMC3172883

