# Supplementary material for: Characterization of Traumatic Brain Injury in a Gyrencephalic Ferret Model Using the Novel Closed Head Injury Model of Engineered Rotational Acceleration (CHIMERA)
Source: Neurotrauma Rep. 2023 Nov 9;4(1):761–80. doi: 10.1089/neur.2023.0047 (PMC10659026; doi:10.1089/neur.2023.0047)

**Supplementary Figure 1:** Representative staining for each region of interest using APP differentiated to white matter structures and subcortical structures. Scale bar = 50µm


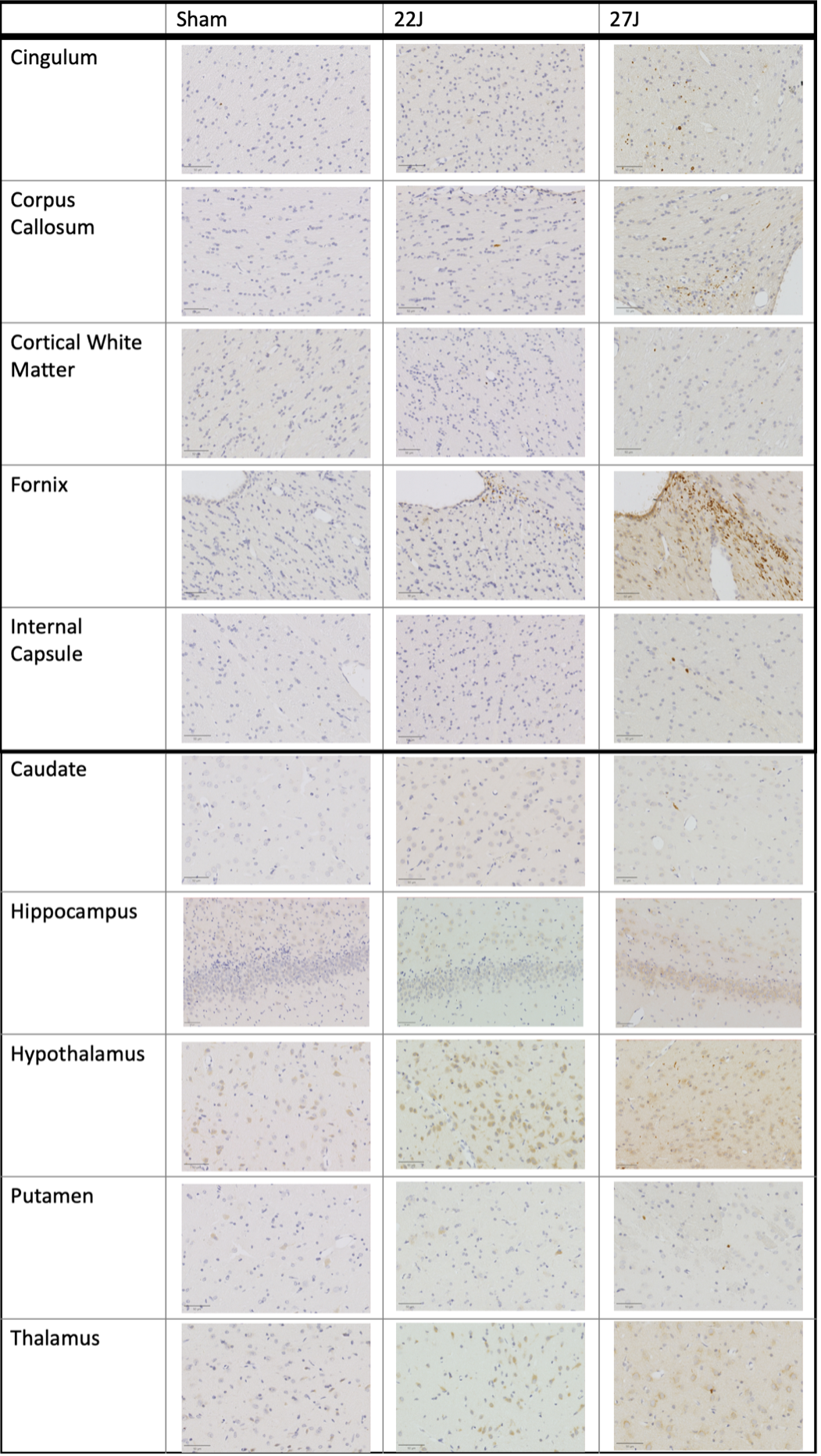

Supplement: Supplemental data [file Suppl_FigureS1.docx]
