# Supplementary figures and images for: Characterization of Traumatic Brain Injury in a Gyrencephalic Ferret Model Using the Novel Closed Head Injury Model of Engineered Rotational Acceleration (CHIMERA)
Source: Neurotrauma Rep. 2023 Nov 9;4(1):761–80. doi: 10.1089/neur.2023.0047 (PMC10659026; doi:10.1089/neur.2023.0047)

**Supplementary Figure 2:** Representative RMO-14 staining for each region of interest (scale bar = 20 µm)


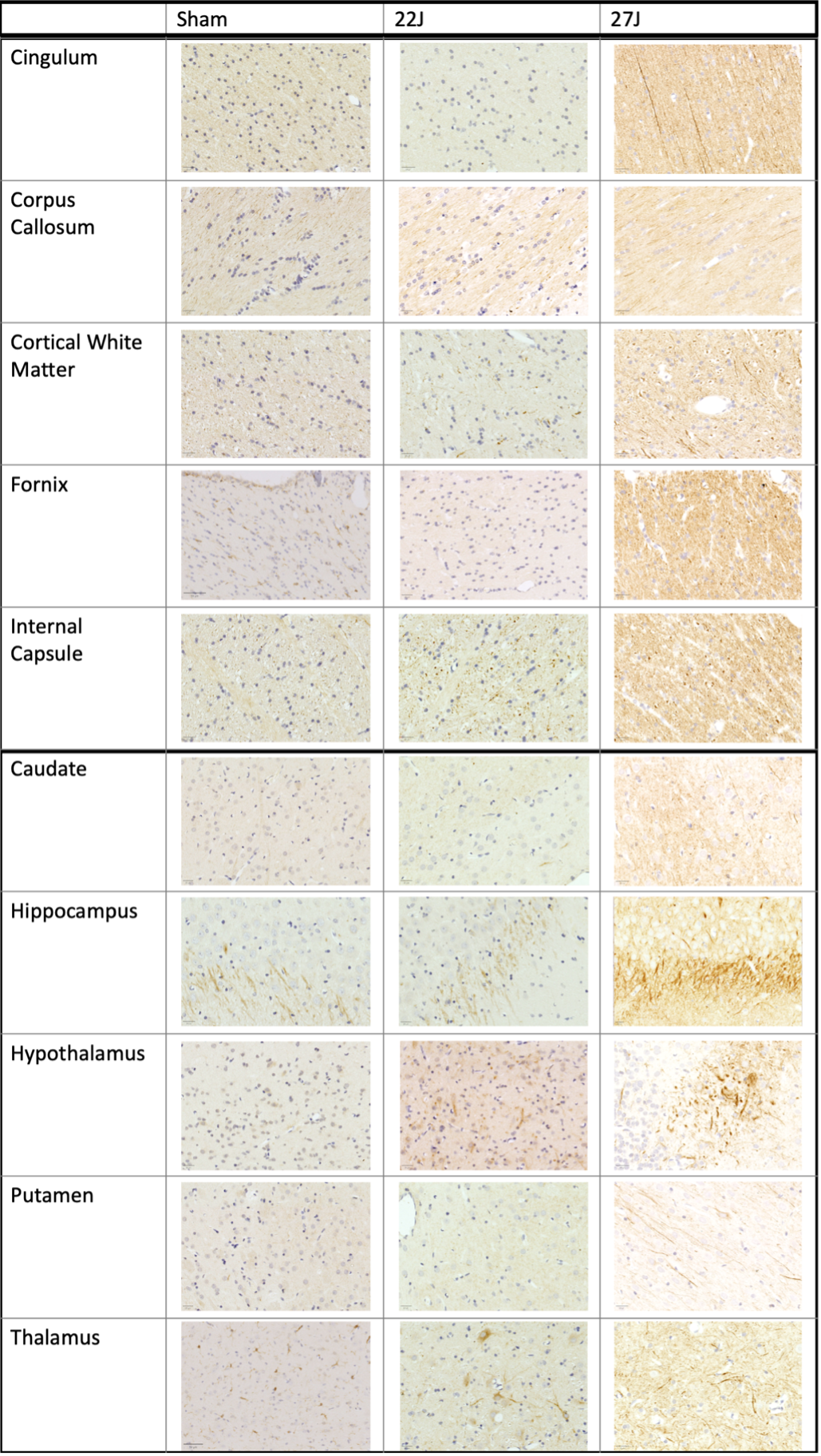

Supplement: Supplemental data [file Suppl_FigureS2.docx]

**Supplementary Figure 3:** Representative IBA-1 staining for each region of interest (scale bar = 50 µm)


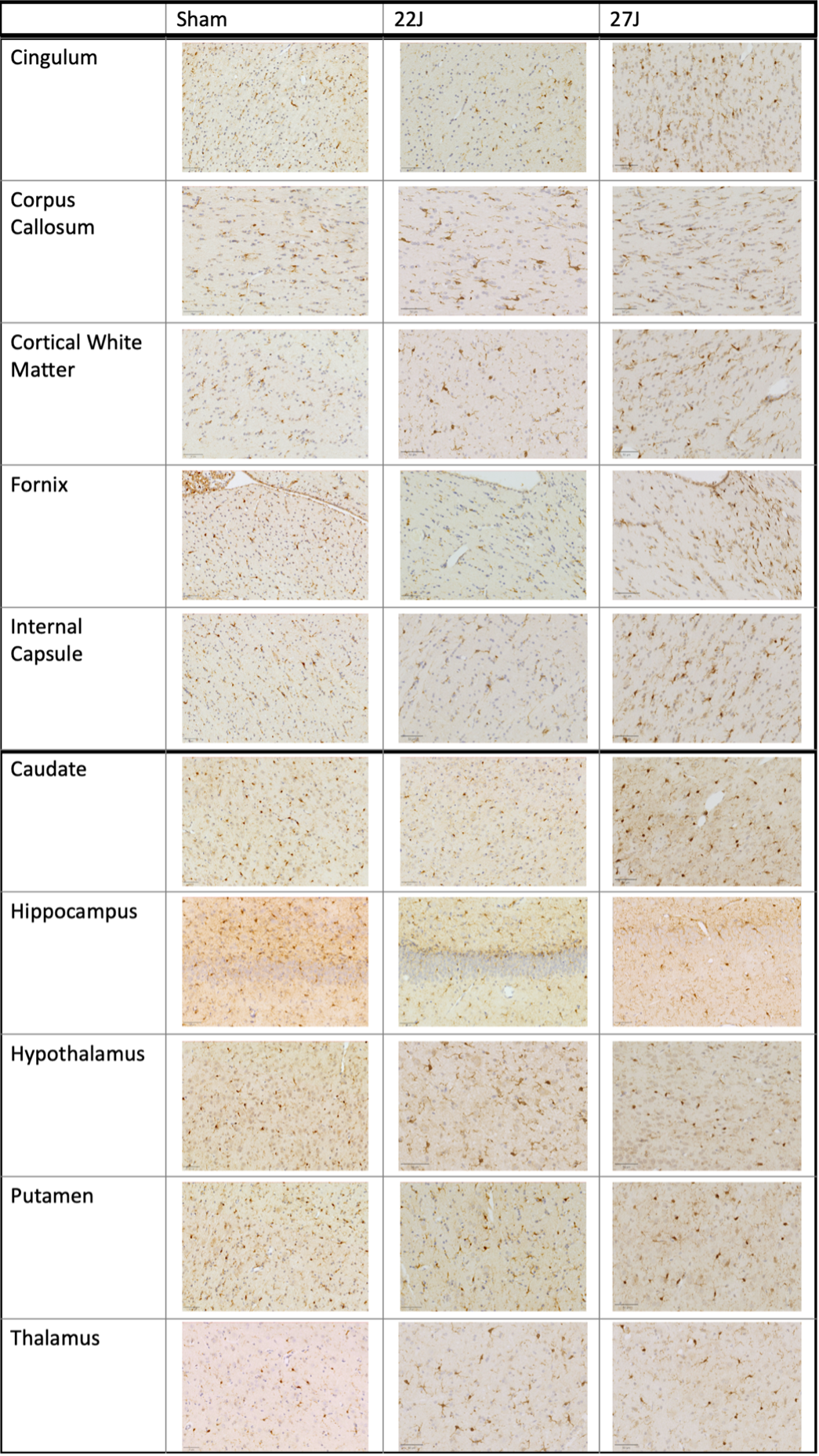

Supplement: Supplemental data [file Suppl_FigureS3.docx]
